# Supplementary material for: SERPINH1 functions as a multifunctional regulator to promote the malignant progression of cervical cancer
Source: PLoS One. 2025 Jul 24;20(7):e0329007. doi: 10.1371/journal.pone.0329007 (PMC12289032; doi:10.1371/journal.pone.0329007)

Marker

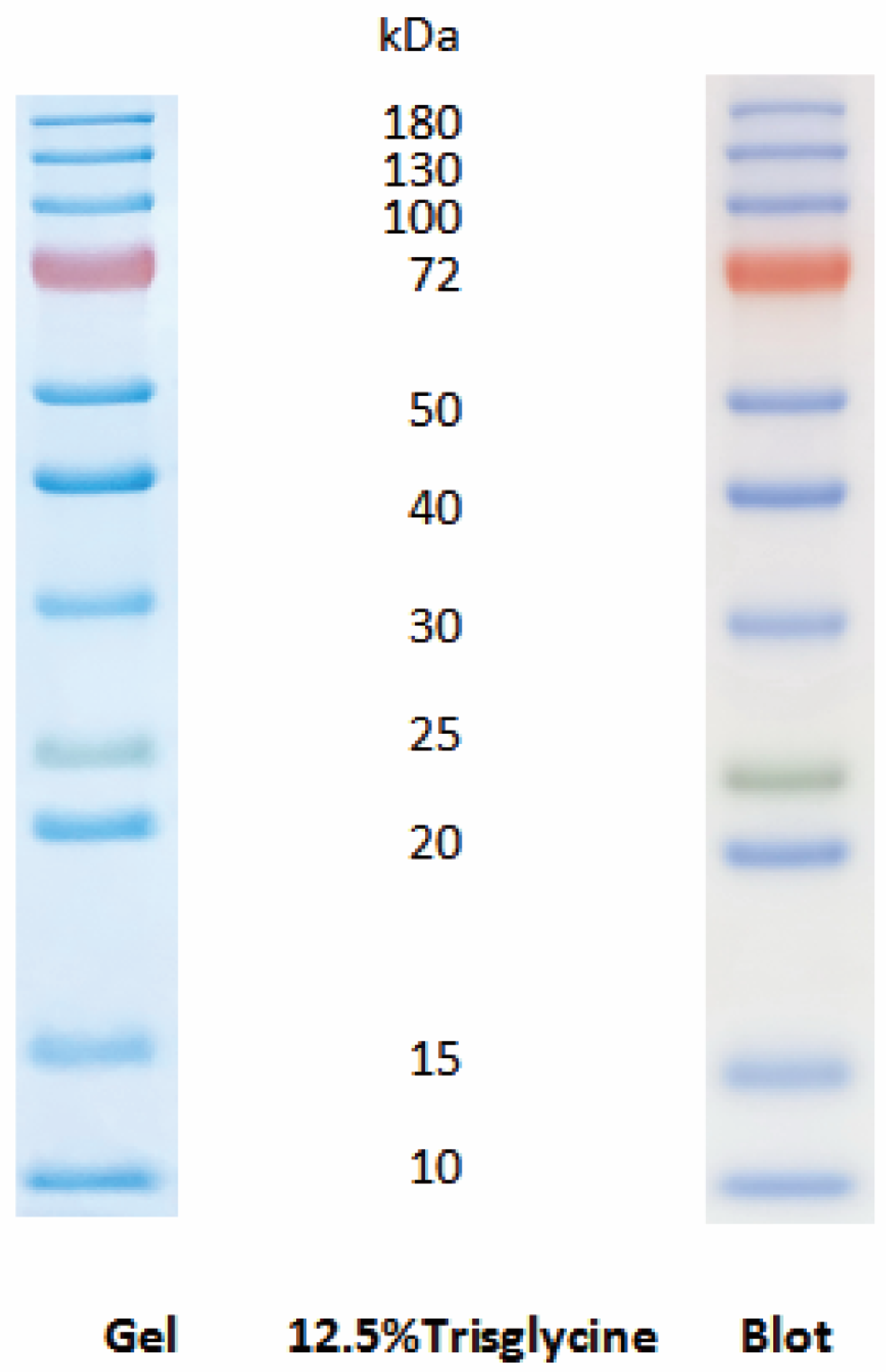

All Western blot images were acquired using the chemiluminescence method with the IMAGEQUANT800 system.

Fig.4A

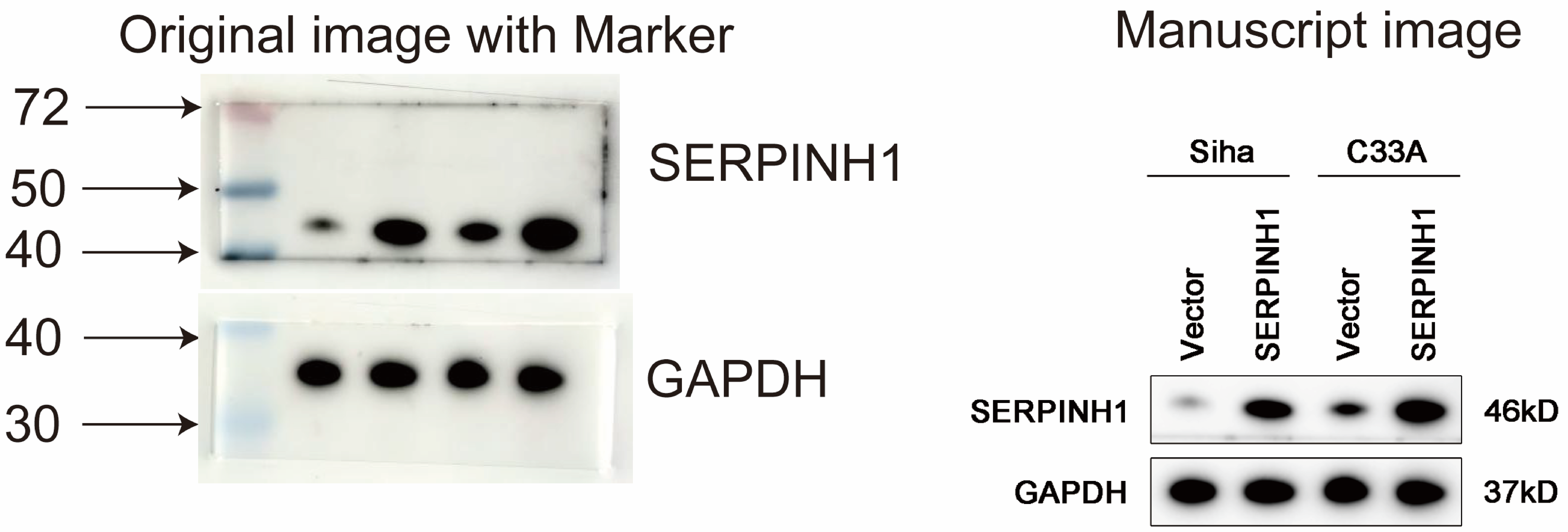

Fig.5A

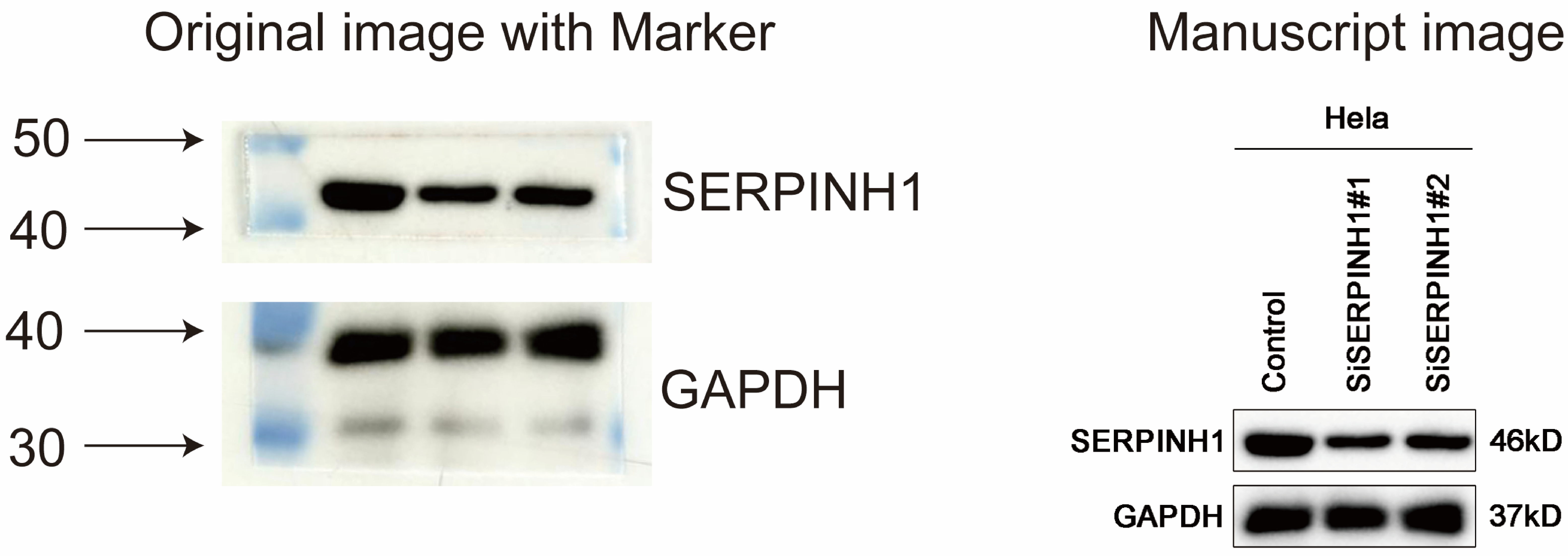

Supplement: S1 File — (PDF) [file pone.0329007.s006.pdf]
