# Supplementary material for: SERPINH1 functions as a multifunctional regulator to promote the malignant progression of cervical cancer
Source: PLoS One. 2025 Jul 24;20(7):e0329007. doi: 10.1371/journal.pone.0329007 (PMC12289032; doi:10.1371/journal.pone.0329007)
Supplement: Attachment — Submitted filename: Point-by-Point Response.docx [file pone.0329007.s008.docx]

**Point-by-Point Response**

**Reviewer #1:** The authors perform several bioinformatic analyses to show the importance of SERPINH1 in the promotion of cervical cancer malignancy. They also perform in vitro assays to support their findings. The manuscript is well written and has important findings. I consider it can be accepted after some minor comments are addressed.

**Response:** Thank you for your careful review and positive comments! We are also grateful for your constructive suggestions, which we believe will significantly improve the clarity and impact of our manuscript.

**Comment 1:** Please specify how many times each experiment was repeated, either in the methods section or in the figure legends.

**Response:** Thanks for the reviewer pointing out this question. All experiments described in this study were independently repeated at least three times. To improve transparency and reproducibility, we have now explicitly stated the number of replicates in the revised ‘Methods’ section and have included this information in the corresponding figure legends.

**Comment 2:** The subfigures of figure 4 are not correctly referred to in the text, please check.

**Response:** Thank you for noticing this inconsistency. We have carefully revised the manuscript to ensure that the subfigures of Figure 4 are correctly referred to in the main text.

**Comment 3:** The authors perform transfection experiments in SiHa and C33 cells, while they do shRNA experiments on HeLa cells. Why were these experiments performed on different cell lines? Please explain in the text.

**Response:** We appreciate the reviewer's insightful question. We have added an explanation in the ‘Methods-Cell culture’ section of the manuscript. Briefly, the choice of different cell lines was based on their histological relevance and biological characteristics to best represent different aspects of cervical cancer. SiHa and C33A are representative of squamous cell carcinoma, while HeLa is an adenocarcinoma cell line, providing a broader understanding of SERPINH1’s effects. Additionally, our preliminary Western blot (WB) validation showed that SERPINH1 expression was relatively highest in HeLa cells, while it was lower in SiHa and C33A cells. This guided our decision to knock down SERPINH1 in HeLa cells and overexpress it in SiHa and C33 cells, thereby providing clearer insights into its role in tumor progression.

**Comment 4:** The abstract states that this study systematically reveals the key role of SERPINH1 as a hub regulator of malignant progression in cervical squamous cell carcinoma (CESC). However, HeLa cell line is an adenocarcinoma cell line and it is also used in this study. Please explain.

**Response:** We appreciate the reviewer for identifying this oversight. We apologize for the misunderstanding caused by the error. The full designation of CESC is Cervical Squamous Cell Carcinoma and Endocervical Adenocarcinoma, but it was incorrectly written as Cervical Squamous Cell Carcinoma (CESC) in the ‘Abstract’. This has been corrected in the revised manuscript. We sincerely thank you for catching this mistake. As previously mentioned, HeLa , SiHa, and C33A cell lines were selected to encompass the major histological subtypes of cervical cancer, thereby allowing a comprehensive assessment of SERPINH1's mechanistic role in cervical carcinogenesis.

**Comment 5:** Section 6. Please define KIRP, LGG, MESO.

**Response:** Thank you for catching this omission. The definitions for KIRP (kidney renal papillary cell carcinoma), LGG (brain lower grade glioma), and MESO (mesothelioma) have been added to Section 6 for clarity.

**Comment 6:** In the abstract, the authors mention the pathways uncovered by GSVA analysis (metabolism, netotic cell death, and alkaliptosis). However, no experiments were performed to validate these findings. Although this is an important finding, this should not be mentioned in the abstract as one of the main findings in the study due to the lack of validation of the pathways.

**Response:** We appreciate the reviewer’s insightful suggestion. We have revised the abstract to remove the reference to GSVA-derived pathways as main findings. Instead, these are now discussed as potential mechanisms warranting further experimental validation.

**Comment 7:** As statistical analysis, the authors perform non-parametric analysis. Did the authors perform a normality test in their data in order to choose non-parametric tests? Please specify.

**Response:** We thank the reviewer for this insightful comment. Our previous description of the statistical analysis methods was incomplete and has now been supplemented. Additionally, normality tests were indeed performed prior to the selection of non-parametric tests, and we have now explicitly mentioned this in the Statistical Analysis section of the manuscript: “Statistical analysis were analyzed using the R software (V.4.3.2, R Core Team, Foundation for Statistical Computing, Vienna, Austria) or GraphPad Prism 9 software (GraphPad Software Inc.). Student’s t-test was used for variables that met the requirements for normal distribution. The Wilcoxon signed-rank test was used for continuous variables that did not meet the requirement of normal distribution. Survival curves were estimated with the Kaplan-Meier method and subsequently compared using log-rank tests. P-value was set at p < 0.05 indicates significance. For all analysis, two-by-two pairs indicate statistically significant differences. *, **, *** and **** indicate, respectively <0.05, <0.01, <0.001, and <0.0001.”

**Reviewer #2:** The manuscript presents a well-structured study with an appropriate methodological design. The topic is relevant, and the findings contribute to the understanding of SERPINH1 in cervical squamous cell carcinoma (CSCC). However, several issues should be addressed to strengthen the manuscript.

**Response:** We appreciate your recognition of the manuscript's relevance and its potential contribution to the field of cervical cancer malignancy! We are also grateful for your constructive suggestions, which we believe will significantly enhance our manuscript.

**Comment 1:** Lack of Supporting References in the Introduction: The authors claim that the specific functions and regulatory mechanisms of SERPINH1 in cervical cancer remain poorly understood. This is an important point, but no references are provided to support this statement. Including relevant citations would substantiate the rationale for the study and contextualize its novelty.

**Response:** We appreciate the reviewer’s valuable suggestion. To strengthen the introduction, we have added relevant citations in paragraph 3 to support the claim that the specific functions and regulatory mechanisms of SERPINH1 in cervical cancer remain poorly understood. These references include recent studies that highlight gaps in current research and substantiate the novelty of our study.

**Comment 2:** Incomplete Literature Review in the Discussion:

**Response:** We appreciate the reviewer pointing out the need for a more comprehensive literature review. We have now incorporated the study by Wang et al. (Front Genet., 2022; doi: 10.3389/fgene.2021.756094), which provides significant insights into the role of SERPINH1 across various cancers: “Wang et al.'s pan-cancer analysis of SERPINH1 revealed its aberrant expression in 14 cancers, with high expression significantly reducing overall survival (OS), disease-specific survival, and progression-free interval in 11 cancers, highlighting its potential as a biomarker (10).” This addition helps contextualize our findings and supports the discussion on SERPINH1’s mechanisms in cervical cancer.

**Comment 3:** Low-Resolution Figures: Figures 2, 3, and 6 are of suboptimal resolution, hindering proper evaluation of the results. The authors are encouraged to replace these with high-quality versions to ensure clarity and reproducibility.

**Response:** Thanks for the reviewer pointing out this question. We have replaced Figures 2, 3, and 6 with high-resolution versions to improve clarity and facilitate proper evaluation.

**Comment 4:** Overstated Conclusions Regarding SERPINH1’s Role: The manuscript proposes that SERPINH1 functions as a central regulator of malignant progression in CSCC via dual modulation of netotic cell death and alkaliptosis. However, the evidence presented does not conclusively support such a definitive role. The authors are advised to temper their conclusions and clearly distinguish between observed data and speculative interpretation.

**Response:** We appreciate the reviewer’s valuable suggestion to refine our conclusions. In response, we have carefully revised the Abstract and Discussion sections to eliminate overstated claims and clearly distinguish between experimental observations and speculative interpretations. Conclusions regarding the regulatory role of SERPINH1 have been moderated to reflect the strength of the data presented. Specifically, the findings related to GSVA-derived pathways are now described as potential mechanisms that suggest biological relevance but warrant further experimental validation. This adjustment ensures that our interpretations remain aligned with the evidence and appropriately cautious: “In summary, our study reveals that SERPINH1 promotes the malignant progression of CESC by enhancing the proliferation and invasion capabilities of CESC cells. Furthermore, enrichment analyses suggest that SERPINH1 may influence tumor malignancy through the regulation of lipid metabolism reprogramming, activation of glycan metabolic pathways, and potential involvement in novel cell death modalities such as NETosis and Alkaliptosis. While these findings provide valuable insights into the multifaceted roles of SERPINH1, further experimental validation is necessary to confirm its regulatory effects on these pathways. Collectively, our study expands the understanding of SERPINH1 as a potential multi-pathway regulator in the malignant progression of CESC, highlighting its potential as a therapeutic target.”
