# Supplementary material for: SERPINH1 functions as a multifunctional regulator to promote the malignant progression of cervical cancer
Source: PLoS One. 2025 Jul 24;20(7):e0329007. doi: 10.1371/journal.pone.0329007 (PMC12289032; doi:10.1371/journal.pone.0329007)
Supplement: Attachment — Submitted filename: Point-by-Point_Response_auresp_2.docx [file pone.0329007.s009.docx]

**Point-by-Point Response**

**Reviewer #1:** The authors have answered my previous comments and I have no further comments. I recommend the manuscript for publication.

**Response:** We are deeply grateful for your time and expertise in reviewing our manuscript! Your constructive feedback during the initial review significantly strengthened our study. Thank you for your final endorsement—we truly appreciate your support in advancing this work.

**Reviewer #3:** This is an interesting and well-written research that is looking for the SERPINH1 role in CESC.

**Response:** Thank you for your careful review and positive comments! We are also grateful for your constructive suggestions, which we believe will significantly improve the clarity and impact of our manuscript.

**Comment 1:**It is not clear why the authors concluded that SERPINH1 is involved in the regulation of lipid metabolism. The authors are overestimating the results, and because of that, the discussion section needs to be modified.

**Response:** We appreciate the reviewer's insightful question. Our claim was based on the following bioinformatic evidence (now explicitly framed as preliminary association in the revised text): In TCGA-CESC cohort analysis (n=304), low SERPINH1 expression tumors showed significant enrichment in lipid metabolism pathways (e.g., LINOLEIC_ACID_METABOLISM: LogFC = -0.62, p<0.000) compared to high SERPINH1 group.

We have revised the ‘Discussion’: These findings revealed an inverse correlation between SERPINH1 expression and activity scores of lipid metabolic pathways. While this suggests a potential link, direct mechanistic evidence for SERPINH1-driven lipid reprogramming requires experimental validation. The clarification of this mechanism could offer novel molecular targets for the development of specific therapeutic strategies aimed at combating metastatic CESC.

**Comment 2:**An interesting result is presented in Fig. 6I, where the authors showed the regulation of the RNA expression levels of ITGA5, PLOD1, and ESM1 by SERPINH1. These results should be better discussed, as this is an event where SERPINH1 expression levels were directly involved and influenced the expression of these genes.

**Response:** We thank the reviewer for highlighting the significance of these findings. As suggested, we have now expanded the discussion of ITGA5, PLOD1, and ESM1 in the revised manuscript (The fourth paragraph of Discussion).

**Comment 3:** Lines 141-145. Determine statistical differences between groups. Here, it is not clear which test was used, as for parametric data the t-test is used, but for non-parametric data the Mann-Whitney U is used. Please clarify and add this information to this section.

**Response:** We thank the reviewer for emphasizing the need for methodological transparency. The statistical analyses have now been explicitly specified in the revised ‘Methods’ section.

**Comment 4:** Line 224. Here it should be Fig. 4B and C

Line 226. The figure reference is wrong; this should be 4D and E

Line 228. Here again, the figure reference is wrong as it should be 4F-I

**Response:** Thank you for carefully checking the figure citations. We have re-examined the original manuscript and confirm that the figure references at these locations were correctly denoted as follows in our submitted version:

Line 224: (Fig 4B-C) revised to (Fig. 4B and 4C)

Line 226: (Fig 4D-E) revised to (Fig. 4D and 4E)

Line 228: (Fig 4F-I) revised to (Fig. 4F-I)

For other  figure references in this manuscript, we have also made corresponding modifications to ensure the uniform format.

**Comment 5:** The researchers transduced SiHa and C33 to overexpress SERPINH1, but the basal protein levels seemed to be high. When the cell lines were transduced, no differences in SERPINH1 levels were observed (Fig. 4A). It would be desirable to carry out a densitometric analysis of this data and to have a quantitative analysis to identify the differences between the vector and the transduced SERPINH1. Also, it would be ideal to determine the percentage of inhibition of SERPINH1 expression after siRNA transfection (Fig. 5A). This is important because siSERPINH1#1 seems to express lower levels of this protein than siSERPINH1#2, and this has different effects on the colony formation, migration, and invasion assays. Revise this and add this information to the text and in the corresponding figure to strengthen the analysis of the results.

**Response:** Thank you for your valuable feedback, which is highly significant for improving our manuscript. To address your concerns, we repeatedly overexpressed SERPINH1 in both Siha and C33A cell lines. The expression levels were detected by Western blotting (WB), and in accordance with your suggestions, we performed quantitative analysis of the WB bands. The results demonstrated that SERPINH1 was upregulated approximately 6-fold in the Siha cell line and approximately 4-fold in the C33A cell line. These data clearly demonstrate a significant difference between the vector control group and the SERPINH1-transfected group (Figure 4A).

In addition, we have quantified the WB bands in Figure 5A and included the percentage inhibition of SERPINH1 expression levels. As shown in the figure, the protein expression level achieved with siSERPINH1#1 was slightly lower than that with siSERPINH1#2, although no statistically significant difference was observed between the two. Furthermore, in subsequent biological functional assays, slight differences between siSERPINH1#1 and siSERPINH1#2 may have arisen due to batch-to-batch variations in transfection efficiency. However, these observed differences fell within statistically acceptable error margins and, crucially, do not affect our conclusion that SERPINH1 promotes the proliferation, migration, and invasion of cervical cancer cells.

Revised Fig. 4A

Revised Fig. 5A

The new Fig. 4A and Fig. 5A have been added to the revised manuscript. We apologize for omitting this key quantification in the original submission.

**Comment 6:** In this part of the experiments to determine the role of SERPINH1 in proliferation, migration, and invasion by knocking down the gene, the researchers used the Hela cells. However, for the previous part of the experiments to show that overexpression stimulates these pathways, the cell lines used were SiHa and C33. There is no explanation for this change. The authors need to address the change of cell lines in the experiments and justify the use of the different cell lines.

On the other hand, the researchers used the HeLa cell line (not transduced), and it is clear from the Western blot that the basal levels of SERPINH1 were high in this cell line (Fig.5A). Why did the authors not use this cell line in all the experiments? How do the basal levels of SERPHIN1 affect or not the results observed?

**Response:** We appreciate the reviewer's question. We have an explanation in the ‘Methods-Cell culture’ section of the manuscript. Briefly, the choice of different cell lines was based on their histological relevance and biological characteristics to best represent different aspects of cervical cancer. SiHa and C33A are representative of squamous cell carcinoma, while HeLa is an adenocarcinoma cell line, providing a broader understanding of SERPINH1’s effects. Additionally, our preliminary Western blot (WB) validation showed that SERPINH1 expression was relatively highest in HeLa cells, while it was lower in SiHa and C33A cells. This guided our decision to knock down SERPINH1 in HeLa cells and overexpress it in SiHa and C33 cells, aiming to induce greater variations in SERPINH1 expression levels within these cell lines, thereby highlighting the impact of this molecule on cancer cells.

**Comment 7:**“SERPINH1 in tumor malignancy through the regulation of lipid metabolism reprogramming, activation of glycan metabolic pathways, and modulation of novel cell death modalities such as NETosis and Alkaliptosis”, the authors are speculating too much about the role of SERPINH1 in regulating these pathways, as this analysis was made through analysis of patients bank genes.

**Response:** We appreciate the reviewer’s insightful suggestion. We have revised the discussion to remove the reference to GSVA-derived pathways as main findings. Instead, these are now discussed as potential mechanisms warranting further experimental validation: Furthermore, GSVA analyses revealed associations between SERPINH1 expression and alterations in lipid metabolism, glycan metabolism pathways, and modulation of novel cell death modalities such as NETosis and Alkaliptosis. While these findings provide valuable insights into the multifaceted roles of SERPINH1, further experimental validation is necessary to confirm its regulatory effects on these pathways.
